# Etiological study of pulmonary infections following solid organ transplantation using metagenomic next-generation sequencing and development of a risk prediction model: a retrospective cohort study

**DOI:** 10.3389/fimmu.2026.1734832

**Published:** 2026-05-28

**Authors:** Hanshui Jiang, Erdan Lu, Qingqing Liu, Zongyu Li, Yan Zhu

**Affiliations:** Department of Pulmonary and Critical Care Medicine, Shulan (Hangzhou) Hospital, Shulan International Medical College, Zhejiang Shuren University, Hangzhou, China

**Keywords:** etiology, prediction model, prognosis, pulmonary infection, solid organ transplantation

## Abstract

**Objective:**

To analyze the pathogenic etiology of pulmonary infection after solid organ transplantation and construct a prognostic prediction model based on metagenomic next-generation sequencing (mNGS) technology, systematically identifying key predictors to provide evidence for clinical risk stratification and individualized interventions.

**Methods:**

Clinical data were retrospectively collected from patients who developed pulmonary infection after liver or kidney transplantation at a single hospital between January 2020 and December 2023. All patients underwent mNGS detection of bronchoalveolar lavage fluid or sputum for pathogen identification. Collected data included demographic characteristics, transplant-related parameters, underlying diseases, laboratory test results, mNGS pathogen detection outcomes, and prognostic indicators. The dataset was randomly divided into a training set (n=262) and a test set (n=66). Within an AutoML framework, model hyperparameters were optimized using the Improved Dharma Optimization Algorithm (IDRA). Feature importance was validated bidimensionally via LASSO regression and SHAP interpretable models, with an interactive MATLAB-based decision support system developed.

**Results:**

The overall positive detection rate of pathogens by mNGS significantly exceeded that of conventional methods (84.76% vs. 61.89%, P<0.001). No statistically significant differences existed in baseline characteristics or laboratory indicators between the training and test sets (all P>0.05), confirming randomized stratified sampling validity. Both cohorts showed highly consistent proportions of poor prognosis events (training set: 27.48% vs. test set: 28.79%, χ^2^=0.045, P = 0.832). The prediction model achieved a ROC-AUC of 0.9694 and PR-AUC of 0.9690 in the training set, and ROC-AUC of 0.9206 (95% CI: 0.854-0.987) with PR-AUC of 0.9273 (95% CI: 0.867-0.988) in the test set, outperforming comparative models. Fourteen key variables were ultimately selected: mNGS bacterial detection, mNGS fungal detection, procalcitonin (PCT), C-reactive protein (CRP), mNGS viral detection, white blood cell count, creatinine, post-transplantation time, neutrophil percentage, diabetes, age, total bilirubin, alanine aminotransferase (ALT), and lymphocyte percentage. The feature overlap rate with AutoML-screened variables was 78.6% (11/14). SHAP analysis revealed descending importance ranking: mNGS bacterial detection, mNGS fungal detection, PCT, etc.

**Conclusion:**

Integrating multidimensional clinical data with explainable machine learning techniques, this study confirms the central role of pathogenic etiology characteristics in prognostic prediction for post-transplant pulmonary infection and demonstrates the potential for real-time risk assessment to inform clinical decisions. However, prospective validation across diverse care settings is required to establish its efficacy as an interventional guide. This work offers innovative tools and methodological frameworks to advance precision diagnosis and management, subject to ongoing refinement through multicenter collaboration.

## 1 Introduction

Solid organ transplantation (SOT) serves as the only effective long-term treatment for patients with end-stage organ failure. With advancements in surgical techniques, optimized immunosuppressive regimens, and enhanced perioperative management, the 1-year survival rate has significantly increased to over 90% (1). However, the immunosuppressed state post-transplantation persistently elevates infection risks, among which pulmonary infection—characterized by insidious symptoms, rapid progression, and diverse pathogens—has emerged as the leading non-neoplastic cause of death in transplant recipients. Clinical data indicate this complication affects 20%–40% of recipients, with mortality exceeding 50% in severe cases, compromising graft function and long-term survival outcomes, thereby representing a critical challenge in transplantation (2, 3).

Conventional pathogenic detection methods (e.g., microbiological culture, microscopy, immunological assays), despite their simplicity and cost-effectiveness, exhibit significant limitations: bacterial cultures require 3–5 days for results, failing to meet the need for rapid early diagnosis; sensitivity for fungi, viruses, and atypical pathogens (e.g., Pneumocystis jirovecii, cytomegalovirus) is hampered by sample quality and culture conditions, resulting in 30%–60% underdiagnosis; furthermore, these methods rarely detect rare pathogens or emerging variants (4, 5). As transplant recipients often present with polymicrobial infections, single conventional tests inadequately capture comprehensive infection profiles, compelling clinicians toward empirical antimicrobial therapy. Such broad-spectrum, blind medication practices may delay optimal treatment, exacerbate antimicrobial resistance, induce drug toxicity, and waste medical resources, ultimately impairing patient recovery (6).

Metagenomic next-generation sequencing (mNGS) offers an innovative diagnostic solution. This hypothesis-free technology comprehensively identifies bacteria, fungi, viruses, and parasites in samples via high-throughput sequencing and bioinformatics analysis within 24–48 hours, simultaneously detecting antibiotic resistance genes and virulence factors for unbiased pathogen screening (7). Current evidence highlights mNGS’s high diagnostic accuracy in immunocompromised cohorts (e.g., hematological malignancies, HIV) (8). Nevertheless, its clinical utility in SOT-associated pulmonary infections warrants further validation. Critically, prognoses among SOT pulmonary infection patients vary substantially, yet no reliable prognostic assessment tool exists. Existing research primarily focuses on etiological diagnosis, with fragmented exploration of prognostic factors, failing to establish an integrated predictive model incorporating clinical features, laboratory indices, and pathogen profiles (9, 10).

Therefore, this study aims to: Systematically evaluate mNGS’s diagnostic value, quantifying its performance against conventional methods and clinical decision-making impact. Identify independent prognostic risk factors and construct/validate a prediction model. These objectives intend to provide theoretical foundations for precision diagnosis and personalized management of post-SOT pulmonary infections.

## 2 Methods

### 2.1 Subjects

**Figure 1:**
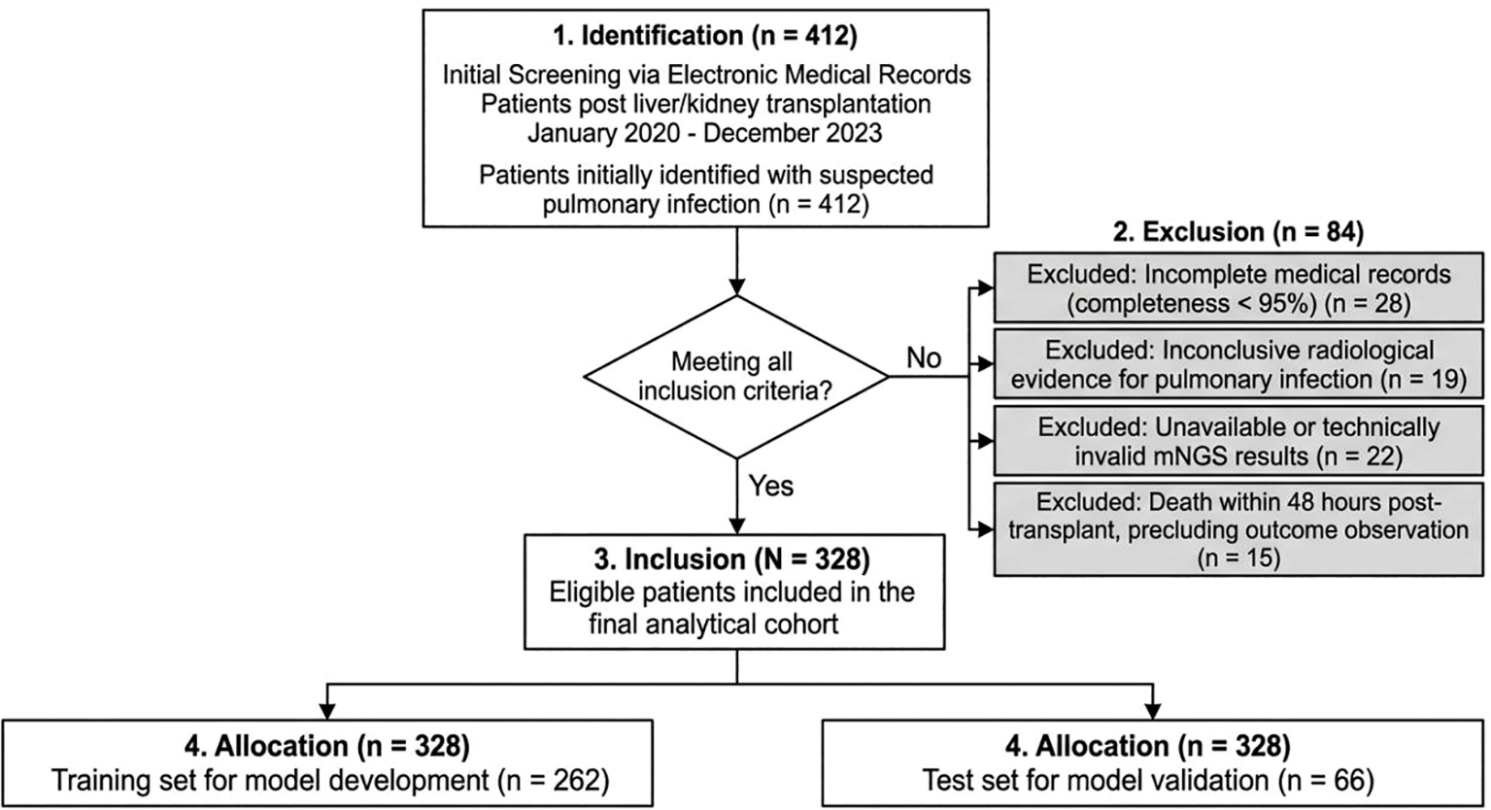
STROBE flowchart.

This study constitutes a retrospective cohort investigation aimed at analyzing the etiological characteristics and prognosis of patients developing pulmonary infections following liver or kidney transplantation. Data were derived from the electronic medical record system of Shulan (Hangzhou) Hospital between January 2020 and December 2023. Throughout the study period, the postoperative infection prevention and management protocols for solid organ transplantation, alongside clinical practices at this institution, remained stable, with no significant alterations in diagnostic or therapeutic pathways that could substantially influence patient prognosis, thereby aiding in reducing confounding biases arising from variations in clinical practice. The participant screening process adhered to the Standards for Reporting of Observational Studies (STROBE) guidelines, with details illustrated in **Figure 1**. Ultimately, a total of 328 patients were included in the final analytical cohort. This study received approval from the hospital ethics committee (Approval No.: KY2025055); informed consent was waived due to the retrospective nature of the research.

Inclusion criteria comprised: (1) Postoperative presentation with definitive pulmonary infection symptoms (e.g., fever, cough, dyspnea) supported by radiological evidence (CT or X-ray); (2) Availability of complete and traceable mNGS results from BALF/sputum samples; (3) Clinical records completeness ≥95%.

Exclusion criteria included: (1) Non-BALF/non-sputum specimens (e.g., blood, throat swabs) or technically invalid mNGS results; (2) Pulmonary infections secondary to extrapulmonary dissemination or indeterminable primary foci; (3) Severe immunodeficiency comorbidities or death within 48 hours post-transplantation precluding outcome observation.

### 2.2 Data collection and processing

Structured electronic medical records provided data on: Demographics: Age, sex, BMI; Transplant parameters: Organ type, post-transplant duration; Comorbidities: Diabetes, hypertension; Laboratory indices: White blood cell count, neutrophil/lymphocyte percentages, platelet count, hemoglobin, creatinine, ALT, AST, total bilirubin, CRP, PCT; mNGS pathogen detection: Viruses, fungi, bacteria; Prognostic indicators.

The composite prognostic endpoint evaluated 30-day outcomes post-infection diagnosis: Poor prognosis: Defined by any of: all-cause mortality, ICU admission, invasive mechanical ventilation, or septic shock. Favorable prognosis: Absence of adverse events with confirmed symptom/cytokine resolution.

Prior to model development, uniform preprocessing was applied to all variables. Continuous variables (such as procalcitonin, age, C-reactive protein, creatinine) retained their original numerical scale without discretization or binning to maximally preserve their predictive information. All continuous variables underwent Z-score standardization (subtracting the mean and dividing by the standard deviation) to eliminate scale effects and accelerate model convergence. Categorical variables (including sex, diabetes status, mNGS pathogen detection results) were transformed into binary dummy variables via one-hot encoding.

The core predictive variables in this study—”mNGS bacterial detection,” “mNGS fungal detection,” and “mNGS viral detection”—were all binary categorical variables. They were defined as follows: Metagenomic Next-Generation Sequencing (mNGS) was performed on bronchoalveolar lavage fluid or sputum samples. After bioinformatics analysis, positivity (coded as 1) was determined if the relative abundance of a specific pathogen (bacteria, fungi, virus) in human-sequence-removed reads exceeded 1% and the number of unique mapped sequences (reads) was ≥3; otherwise, it was classified as negative (coded as 0). This threshold integrated considerations of test sensitivity, background noise, and clinical relevance, representing one of the current standard criteria for clinical interpretation of mNGS pathogen detection.

This study followed a transparent and reproducible predictive model development workflow in compliance with the TRIPOD-AI reporting guidelines. For model validation, the final cohort of 328 patients was randomly partitioned into training and independent test sets at an approximate 4:1 ratio (training set: n=262; test set: n=66). This partitioning ratio is widely adopted in machine learning practice to provide sufficient samples for model training while reserving an adequate number of independent samples for unbiased validation. Crucially, the randomization was stratified based on the “prognostic event” (i.e., poor prognostic outcomes) to ensure consistent incidence rates of adverse outcomes between training and test sets. This prevented selection bias from data splitting and ensured equitable model performance evaluation.

To comprehensively evaluate data quality and ensure analytical robustness, we quantified missing data proportions for all included variables (detailed in **Supplementary Material 1**). Overall data completeness was high, with most variables having <5% missingness. Differential imputation strategies were implemented based on missingness patterns and variable importance: For categorical variables with minimal missingness (<2%) lacking explicit clinical stratification (e.g., sex), mode imputation was used; for categorical variables clinically linked to prognosis or exposure (e.g., diabetes status, mNGS pathogen detection), inference imputation followed clinical logic or group probabilities. For continuous variables with strong linear correlations to another variable (e.g., hemoglobin vs. hematocrit), regression imputation was applied; otherwise, stratified imputation based on clinically relevant subgroups (e.g., transplant organ type) used subgroup-specific mean or median imputation. For critical continuous prognosis predictors (e.g., procalcitonin, creatinine), multiple imputation was additionally employed to quantify missing data uncertainty. Specifically, 20 complete datasets were generated using the chained equations method. Models were developed and evaluated separately on each dataset, and final model parameters/performance metrics were pooled via Rubin’s rules, providing point and interval estimates accounting for missing data variability.

**Figure 2:**
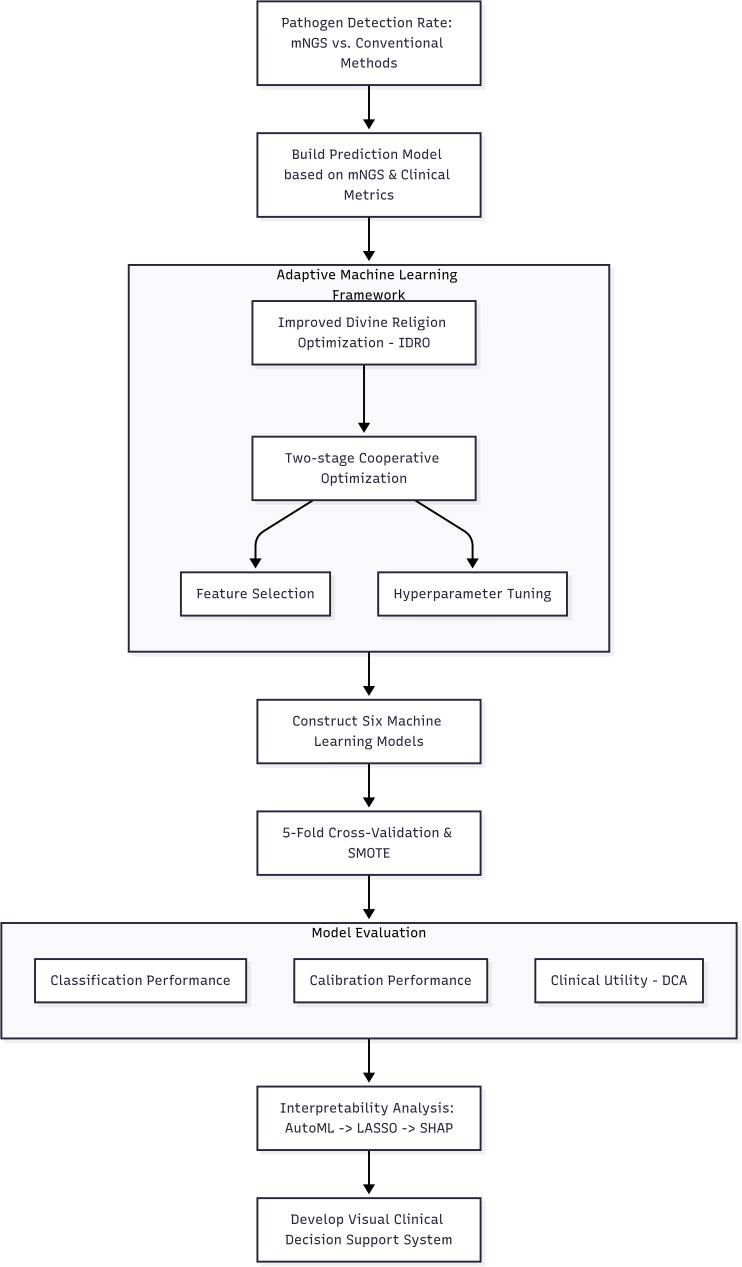
Technical workflow.

Diagnostic efficiency of mNGS versus conventional methods (including BALF-based smear/culture) was compared, followed by prognostic model construction integrating mNGS findings with clinical variables (see **Figure 2** for technical workflow).

### 2.3 Sample size rationality assessment

This retrospective observational study aimed to develop and validate a prognostic prediction model. The sample size determination primarily relied on the total number of eligible cases meeting inclusion and exclusion criteria during the study period (N = 328). To avoid overfitting in machine learning models, a widely accepted empirical rule was followed—ensuring adequate Events Per Variable (EPV). In this study, the training set (n=262) included 72 poor prognosis events. After feature selection, 11 predictor variables were retained in the final model (based on the AutoML-screened core feature set). Consequently, the EPV reached approximately 6.5 (72 events/11 variables), which falls below the ideal threshold of EPV ≥10 commonly recommended in prediction model studies. However, it exceeds the minimum accepted threshold of EPV ≥5, indicating that the sample size is fundamentally reasonable relative to model complexity and helps mitigate overfitting risks. Nevertheless, we acknowledge that a larger sample size would further enhance the model’s stability and generalizability, which will be addressed in the study limitations section.

### 2.4 Automated machine learning

This study established an Automated Machine Learning (AutoML) framework based on the Improved Divine Religions Algorithm (IDRA), designed to automate the dual tasks of feature selection and model hyperparameter optimization. The core optimization workflow of this framework involves: Phase one screens high-weight feature subsets in discrete space; Phase two fine-tunes model hyperparameters in continuous space.

Throughout model development, we rigorously implemented multiple strategies to mitigate overfitting. First, all models were developed and tuned via five-fold cross-validation exclusively on the training set. Specifically, the training set was further randomly divided into five similarly-sized subsets; each iteration used four subsets for training and one for validation, cycling through all five subsets. The training set performance metrics reported in this study represent the average results of five-fold cross-validation. This approach provides a more robust estimate of the model’s true generalization ability compared to single training-set evaluations. Second, within the AutoML framework, the IDRA algorithm prevents overfitting to training data through regularization constraints and early stopping strategies based on validation set performance.

Following automated search and optimization by the AutoML framework, the optimal base prediction model architecture was designated the “AutoML model.” For fair comparison, we concurrently constructed five traditional machine learning models as baselines: Logistic Regression (LR), Support Vector Machine (SVM), Adaptive Boosting (AdaBoost), Extreme Gradient Boosting (XGBoost), and Light Gradient Boosting Machine (LightGBM). To ensure comparison fairness and reproducibility, all comparator models (including LR, SVM, etc.) underwent identical data preprocessing procedures (covering standardization, missing value handling, etc.) as the AutoML model. Furthermore, each model independently underwent hyperparameter grid search optimization via the same five-fold cross-validation process on the training set to obtain their respective optimal parameter combinations, thereby avoiding performance bias stemming from differential preprocessing or tuning depths.

### 2.5 Evaluation metrics

A multidimensional assessment system was established: (1) Classification performance: Prognosis prediction models were evaluated using accuracy (ACC), sensitivity (SEN), specificity (SPE), F1-score (harmonic mean of precision and recall), area under the receiver operating characteristic curve (AUC-ROC), and area under the precision-recall curve (AUC-PR) to systematically quantify discriminative power and stability under class imbalance. (2) Calibration performance: Model calibration performance was evaluated using calibration curves and the Brier score. The calibration process did not employ additional post-processing techniques (such as Platt scaling or isotonic regression). Consequently, the calibration curve directly reflects the raw calibration characteristics of the model’s output probabilities, particularly those of the finally selected AutoML gradient boosting tree model. The Brier score (where lower values indicate greater accuracy in probabilistic prediction) was used to quantify the calibration error. (3) Clinical utility: Decision curve analysis (DCA) quantified net benefit (NB) across threshold probabilities, where NB = (TP/N) – (FP/N) × (pt/(1–pt)), with TP/FP denoting true/false positives, N the sample size, and pt the risk threshold. NB values were benchmarked against traditional intervention strategies to identify clinically actionable ranges.

### 2.6 Interpretability analysis

Following initial feature screening via AutoML, robustness was verified through LASSO regression, and clinical rationality was examined using SHAP interpretability. The workflow comprised: (1) AutoML-based preliminary screening: Automatically identifying prognosis-associated feature subsets within predefined search spaces; (2) LASSO verification: Applying L1 regularization to validate feature sparsity and stability against overfitting, while comparing differences between LASSO and AutoML outputs; (3) SHAP analysis: Quantifying feature contributions through cooperative game theory, visualizing global feature importance rankings to rationalize prediction logic.

### 2.7 Statistical methods

Data were processed in SPSS 26.0. Normally distributed continuous variables were expressed as mean ± standard deviation ( $\bar{\text{x}}\pm \text{s}$), non-normal variables as median [interquartile range] [M(IQR)], and categorical variables as frequency [percentage] [n(%)]. Intergroup comparisons included: one-way t-tests for normally distributed continuous variables; Mann-Whitney U tests for non-normal distributions; Pearson’s chi-square tests for categorical variables. Statistical significance was defined as two-sided P<0.05, with results presented in structured tables.

## 3 Results

### 3.1 Baseline characteristics of the study population

Among the 328 patients included in the analysis, the data completeness of all variables was high. As shown in **Table 1**, with the exception of a few laboratory indicators (such as procalcitonin and C-reactive protein) which had approximately 5-8% missing data due to issues with testing timing, other demographic, underlying disease, and mNGS core variables had missing rates below 2%. Targeted data imputation was performed according to predefined strategies, with specific details available in **Supplementary Material 1**. For key variables processed using multiple imputation, the final analysis results incorporated the uncertainty introduced by imputation.

**Table 1:** Comparison of baseline characteristics between training and test sets.

Feature	Overall (n=328)	Training set (n=262)	Test set (n=66)	Statistic	P-value
Poor outcome events, *n* (%)	91 (27.74%)	72 (27.48%)	19 (28.79%)	χ^2^ = 0.045	0.832
Age (years), Mean ± SD	50.61 ± 10.72	50.32 ± 10.56	51.78 ± 11.32	t = 0.989	0.323
Male sex, *n* (%)	202 (61.59%)	157 (59.92%)	45 (68.18%)	χ^2^ =1.520	0.218
BMI (kg/m^2^), Median [IQR]	24.10 [22.00-26.10]	24.00 [22.00-26.00]	24.50 [22.00-26.50]	Z = 0.234	0.815
Transplant Organ Type, n (%)				χ^2^ = 0.349	0.555
Liver	262 (79.88%)	211 (80.53%)	51 (77.27%)	–	–
Kidney	66 (20.12%)	51 (19.47%)	15 (22.73%)	–	–
Time Post-Transplant (months), Median [IQR]	12.10 [6.00-24.20]	12.00 [6.00-24.00]	12.50 [6.00-25.00]	Z = 0.123	0.902
Diabetes, *n* (%)	88 (26.83%)	68 (25.95%)	20 (30.30%)	χ^2^ = 0.508	0.476
Hypertension, *n* (%)	165 (50.30%)	127 (48.47%)	38 (57.58%)	χ^2^ = 0.774	0.379
White Blood Cell Count (x10^9/L), Mean ± SD	10.27 ± 2.80	10.19 ± 2.75	10.57 ± 2.99	t = 0.986	0.325
Neutrophil Percentage (%), Mean ± SD	75.96 ± 5.57	75.73 ± 5.42	76.86 ± 6.11	t = 1.475	0.141
Lymphocyte Percentage (%), Mean ± SD	19.95 ± 5.76	20.16 ± 5.92	19.14 ± 5.01	t = 1.288	0.199
Platelet Count (x10^9/L), Mean ± SD	215.81 ± 51.93	217.34 ± 52.45	209.73 ± 49.73	t = 1.064	0.288
Hemoglobin (g/L), Mean ± SD	120.20 ± 10.20	120.00 ± 10.00	121.00 ± 11.00	t = 0.500	0.617
Creatinine (μmol/L), Median [IQR]	101.01 [80.40-121.01]	100.00 [80.00-120.00]	105.00 [82.00-125.00]	Z = 0.345	0.730
Alanine Aminotransferase (U/L), Median [IQR]	40.40 [30.40-50.40]	40.00 [30.00-50.00]	42.00 [32.00-52.00]	Z = 0.267	0.789
Aspartate Aminotransferase (U/L), Median [IQR]	35.20 [25.20-45.20]	35.00 [25.00-45.00]	36.00 [26.00-46.00]	Z = 0.312	0.755
Total Bilirubin (μmol/L), Median [IQR]	15.20 [10.20-20.20]	15.00 [10.00-20.00]	16.00 [11.00-21.00]	Z = 0.189	0.850
C-Reactive Protein (mg/L), Median [IQR]	20.20 [10.20-30.20]	20.00 [10.00-30.00]	21.00 [11.00-31.00]	Z = 0.423	0.672
Procalcitonin (ng/mL), Median [IQR]	0.51 [0.21-1.01]	0.50 [0.20-1.00]	0.55 [0.25-1.05]	Z = 0.501	0.616
mNGS Virus Detected, n (%)	98 (29.88%)	78 (29.77%)	20 (30.30%)	χ^2^ = 0.007	0.933
mNGS Fungus Detected, n (%)	87 (26.52%)	69 (26.34%)	18 (27.27%)	χ^2^ = 0.024	0.878
mNGS Bacteria Detected, n (%)	196 (59.76%)	156 (59.54%)	40 (60.61%)	χ^2^ = 0.025	0.875

No significant statistical differences were observed in baseline characteristics or laboratory indices between the training set (n=262) and test set (n=66), confirming the effectiveness of randomized stratified sampling (all P>0.05). The proportions of poor prognosis events were highly consistent across both sets (training set: 27.48% vs. test set: 28.79%; χ^2^=0.045, P = 0.832).

The composite endpoint “poor prognosis” in this study comprised four components. Among the total 328 patients, there were 91 occurrences of adverse prognosis events (some patients experienced multiple events). The specific event counts and proportions for each component are presented in **Table 2**. Among them, ICU admission was the most common event, followed by death and the requirement for invasive mechanical ventilation.

**Table 2:** Event distribution of components in the composite endpoint “poor prognosis”.

Endpoint component	Event count (n)	% of total patients	% of patients with poor prognosis*
All-cause death	22	6.71%	24.18%
ICU admission	54	16.46%	59.34%
Invasive mechanical ventilation required	31	9.45%	34.07%
Septic shock	18	5.49%	19.78%
Any poor prognosis (Composite endpoint)	91	27.74%	100.00%

The denominator for this column is the total number of patients experiencing any poor prognosis (n=91). Percentages sum to >100% as individual patients may experience multiple events.

### 3.2 Comparison of pathogen detection rates between mNGS and conventional microbiology methods

The findings revealed that metagenomic next-generation sequencing (mNGS) demonstrated a significantly higher overall positive detection rate for pathogens in pulmonary infections among solid organ transplantation recipients compared to conventional methods (84.76% vs. 61.89%, P<0.001). mNGS exhibited particularly prominent advantages in detecting viruses (29.88% vs. 12.80%, P<0.001) and fungi (26.52% vs. 15.55%, P = 0.001), while its bacterial detection rate also surpassed that of traditional culture methods (59.45% vs. 51.22%, P = 0.034). Moreover, mNGS identified a significantly higher proportion of mixed infections (22.26% vs. 8.54%, P<0.001). Detailed results are presented in **Table 3**.

**Table 3:** Comparison of pathogen detection rates between mNGS and conventional methods (n=328).

Pathogen type	mNGS detection count (%)	Conventional method detection count (%)	Absolute difference (95% CI)	*χ*^2^-value	*P*-value
Virus	98 (29.88%)	42 (12.80%)	17.07% (10.87% to 23.14%)	28.478	<0.001
Cytomegalovirus (CMV)	65 (19.82%)	28 (8.54%)			
Epstein-Barr Virus (EBV)	45 (13.72%)	12 (3.66%)			
Human Herpesvirus-6 (HHV-6)	18 (5.49%)	5 (1.52%)			
Respiratory Syncytial Virus (RSV)	22 (6.71%)	15 (4.57%)			
Fungi	87 (26.52%)	51 (15.55%)	10.98% (4.75% to 17.11%)	11.893	0.001
Pneumocystis jirovecii (PJP)	38 (11.59%)	12 (3.66%)			
Aspergillus spp	35 (10.67%)	18 (5.49%)			
Cryptococcus spp	10 (3.05%)	8 (2.44%)			
Candida spp	25 (7.62%)	22 (6.71%)			
Bacteria	195 (59.45%)	168 (51.22%)	8.23% (0.63% to 15.70%)	4.496	0.034
Pseudomonas aeruginosa	58 (17.68%)	52 (15.85%)			
Klebsiella pneumoniae	49 (14.94%)	47 (14.33%)			
Legionella pneumophila	15 (4.57%)	6 (1.83%)			
Haemophilus influenzae	32 (9.76%)	25 (7.62%)			
Streptococcus pneumoniae	28 (8.54%)	22 (6.71%)			
Anaerobes (mixed)	20 (6.10%)	18 (5.49%)			
Mixed Infections(≥2)	73 (22.26%)	28 (8.54%)	13.72% (8.26% to 19.17%)	23.698	<0.001
Overall	278 (84.76%)	203 (61.89%)	22.87% (16.21% to 29.26%)	43.837	<0.001

### 3.3 Performance evaluation of improved swarm intelligence algorithm

**Figure 3:**
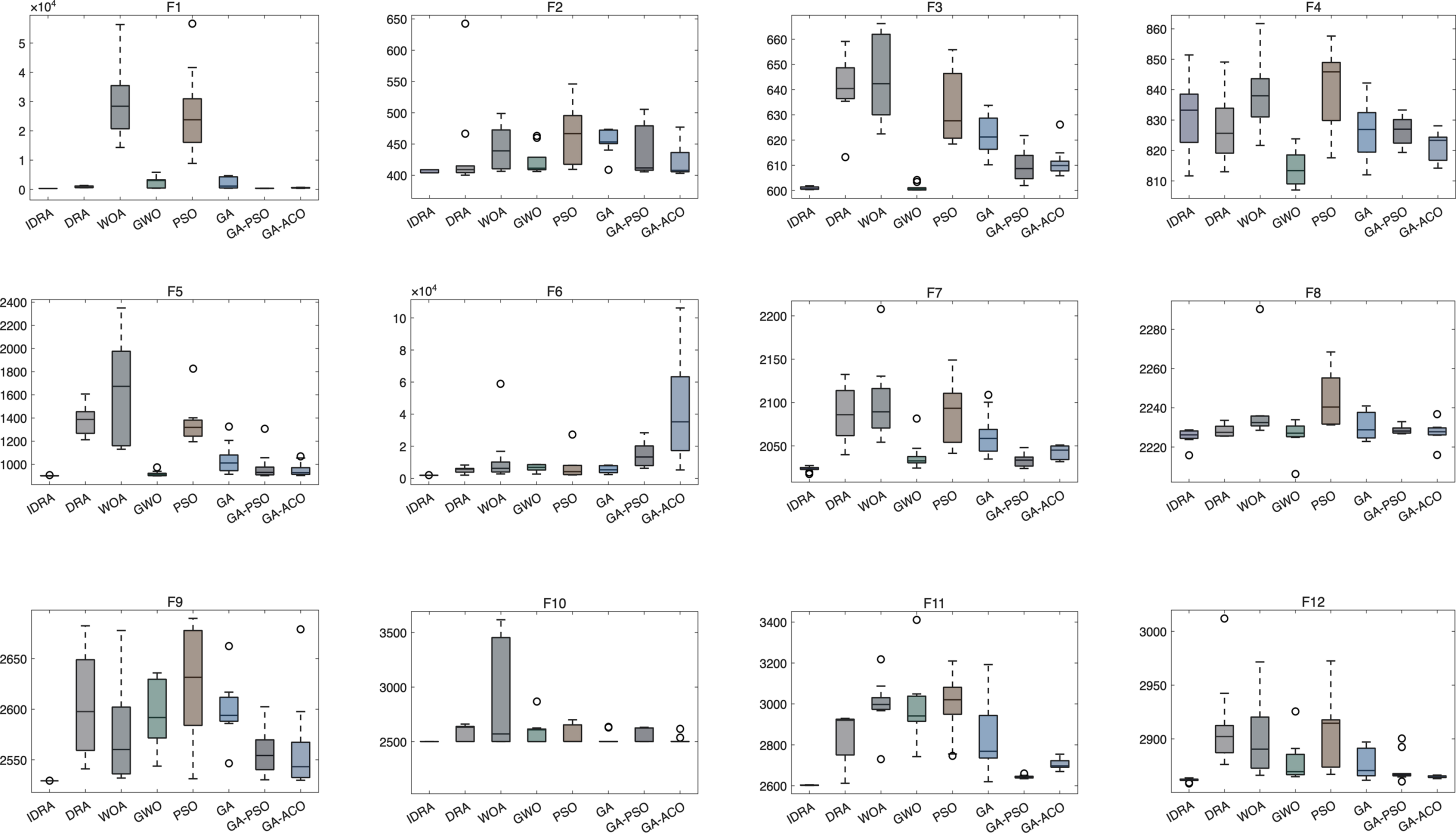
Optimization performance comparison of swarm intelligence algorithms. Comparison of solution stability across different algorithms on each test function. This panel consists of 12 subplots corresponding to the 12 CEC2022 benchmark functions (F1–F12). In each subplot, the x-axis represents the eight algorithms compared (IDRA, DRA, WOA, GWO, PSO, GA, GA-PSO, GA-ACO), and the y-axis represents the best fitness value (optimal solution) obtained from 30 independent runs. The box plots illustrate the distribution and stability of the solutions obtained by each algorithm on a specific function.

**Figure 4:**
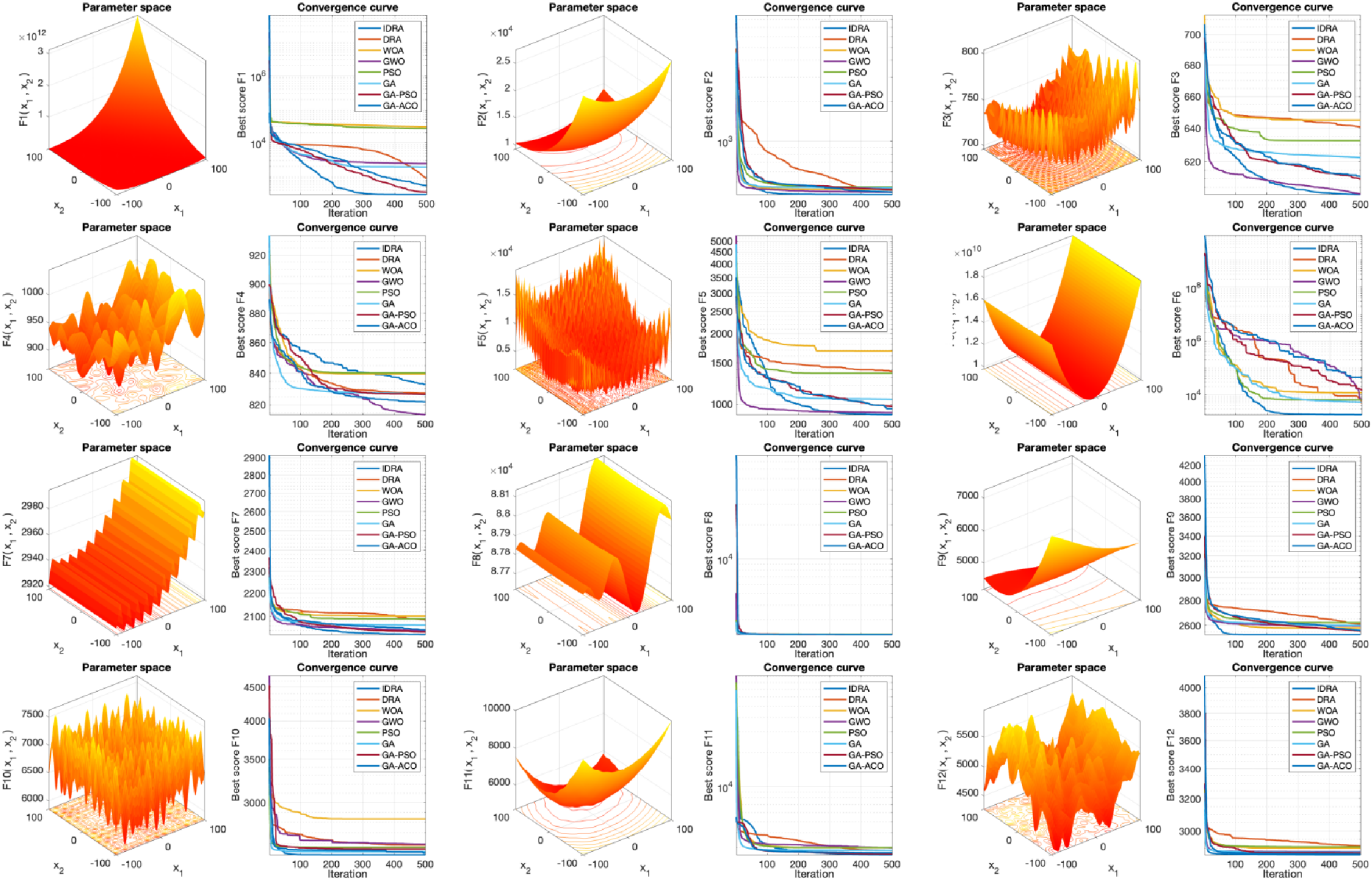
Convergence performance comparison of swarm intelligence algorithms. Comparison of convergence curves across different algorithms on each test function. This panel consists of 12 subplots corresponding to the 12 CEC2022 benchmark functions (F1–F12). In each subplot, the x-axis represents the iteration number (1–500), and the y-axis represents the best fitness value of the current population (logarithmic scale). Each curve depicts the evolution of the best solution found by an algorithm as the iterations proceed. Experimental settings: All tests were conducted with a dimension of 10, a population size of 30, and a maximum of 500 iterations. The results shown are statistical summaries from 30 independent runs.

To verify the optimization capability of the improved IDRA algorithm, this study conducted comparative tests against the original DRA, WOA, GWO, PSO, GA, GA-PSO, and GA-ACO algorithms. The experiment employed all 12 benchmark functions from the CEC2022 test set, with the variable dimension of all test functions set to 10, population size to 30, and maximum iterations to 500. Each algorithm was independently run 30 times to ensure statistical reliability. By comparing the 30 runs, box plots were generated to evaluate the optimization stability of each algorithm. Results demonstrated that IDRA outperformed others on most test functions, exhibiting significantly superior stability compared to the original DRA and other algorithms (**Figure 3**). Further analysis of convergence curves revealed that IDRA achieved faster convergence speed with the lowest risk of falling into local optima during iterations (**Figure 4**). These experimental results fully confirm IDRA’s significant advantages in global optimization performance and convergence efficiency.

### 3.4 Model training evaluation

**Figure 5:**
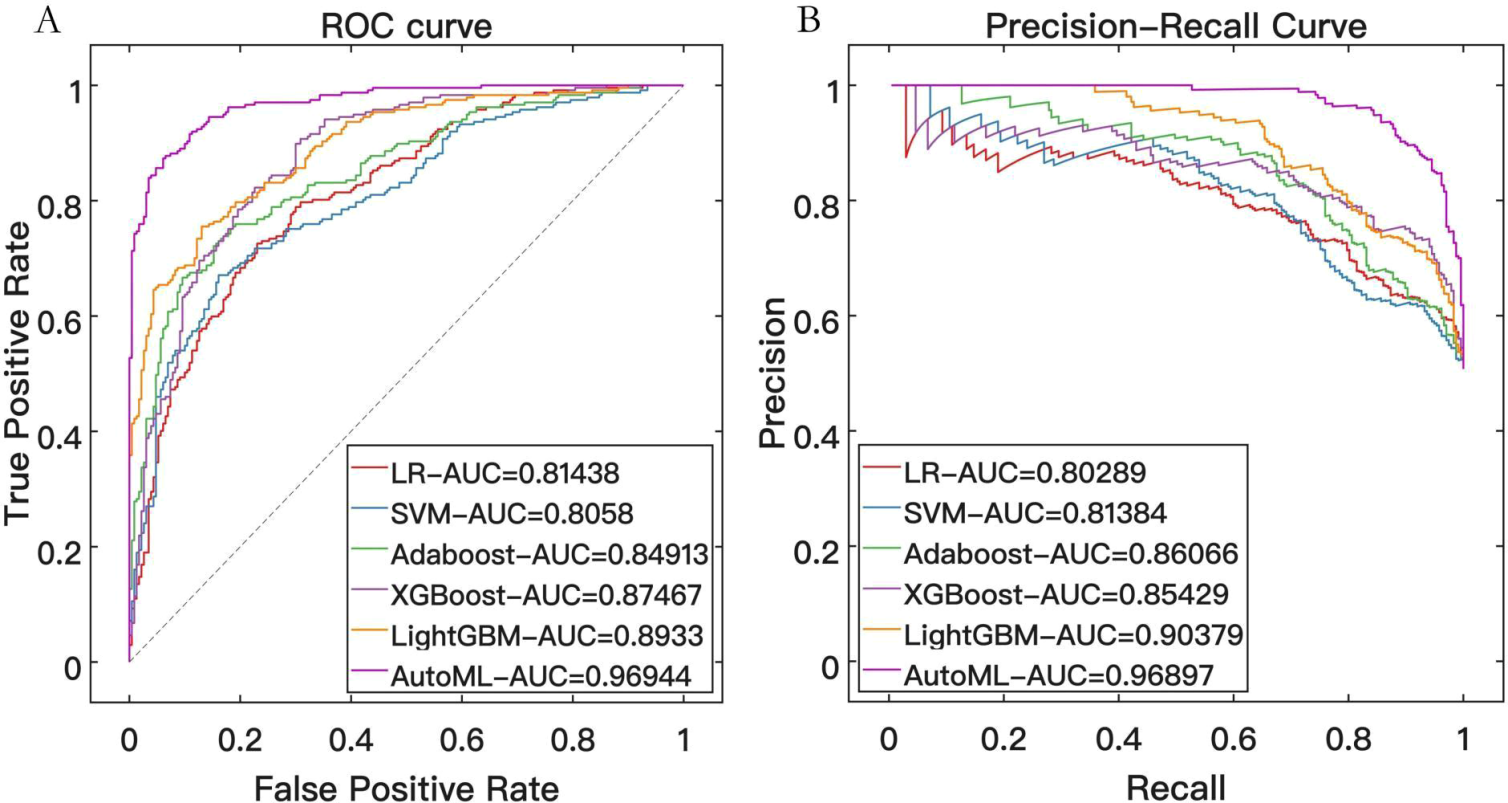
Training set cross-validation performance. **(A)** Training set ROC curve; **(B)** Training set PR curve.

After automated search and optimization via the AutoML framework, the final optimal base predictive model architecture was identified as a variant of gradient boosting trees. Its key hyperparameters, optimized using the Improved Dharma Optimization Algorithm (IDRA), were set as follows: learning rate = 0.05, max tree depth = 6, subsampling proportion = 0.8, proportion of features used in modeling = 0.9, number of trees (iterations) = 150. This model was designated as the “AutoML model”. This study systematically assessed the predictive performance of six machine learning models on the training set using metrics including precision (PRE), sensitivity (SEN), specificity (SPE), accuracy (ACC), F1-score, and area under the curve (AUC). Results showed that the AutoML model delivered comprehensively optimal performance, with ROC-AUC reaching 0.9694 and PR-AUC 0.9690. Notably, AutoML’s advantage was particularly prominent in the F1-score (0.8964), indicating its greater clinical utility in balancing precision-recall tradeoffs. AutoML ultimately identified the following features: mNGS bacterial detection, mNGS fungal detection, Procalcitonin (PCT), C-reactive protein (CRP), mNGS viral detection, white blood cell count, creatinine, post-transplantation time, neutrophil percentage, diabetes, and age. For details, see **Table 4** and **Figure 5**.

**Table 4:** Training set cross-validation predictive performance metrics.

Models	PRE	SEN	SPE	ACC	F1	ROC-AUC	PR-AUC
LR	0.5609	0.9916	0.2000	0.6017	0.7165	0.8144	0.8029
SVM	0.5859	0.9494	0.3087	0.6338	0.7246	0.8058	0.8138
Adaboost	0.7290	0.8059	0.6913	0.7495	0.7655	0.8491	0.8607
XGBoost	0.6706	0.9620	0.5130	0.7409	0.7903	0.8747	0.8543
LightGBM	0.7285	0.8945	0.6565	0.7773	0.8030	0.8933	0.9038
AutoML	0.8491	0.9494	0.8261	0.8887	0.8964	0.9694	0.9690

### 3.5 Test set predictive performance comparison

**Figure 6:**
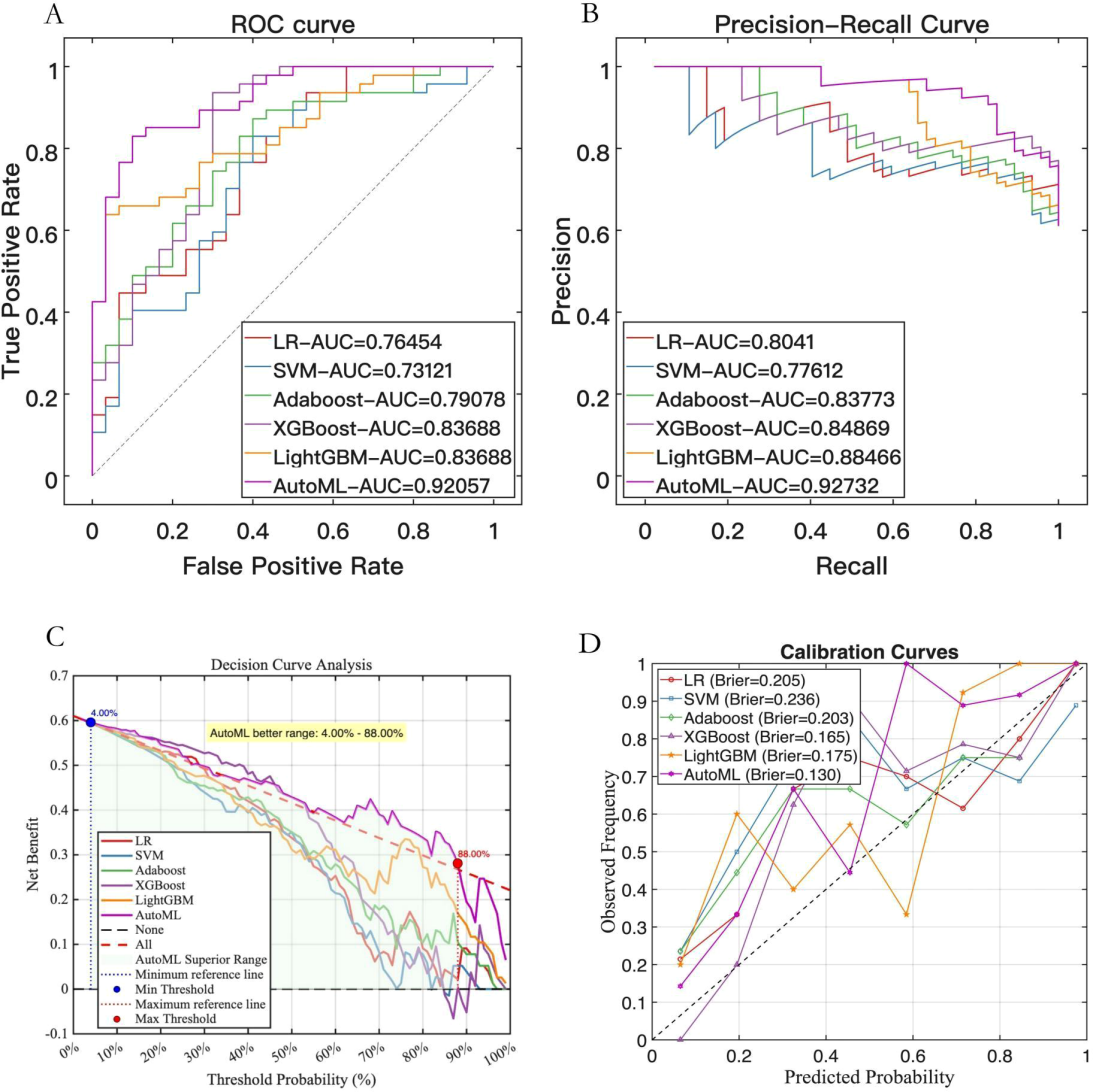
Test set predictive performance evaluation. **(A)** Test set ROC curve; **(B)** Test set PR curve; **(C)** Test set DCA curve; **(D)** Test set calibration curve.

This study systematically evaluated the performance of six machine learning models on the test set for predicting the prognosis of pulmonary infections after solid organ transplantation. Results demonstrated that: AutoML exhibited the strongest robustness in the independent test set, achieving ROC-AUC of 0.9206 and PR-AUC of 0.9273 (**Figures 6A, B**). The DCA demonstrated significant net benefit gains for clinical intervention guided by our prognostic model (**Figure 6C**). Quantitatively, our model outperformed both “treat-all” and “treat-none” strategies across the clinically critical threshold range of 15%–60% predicted risk. Within this interval, the net benefit attained a maximum value of, indicating that utilizing the model to guide clinical interventions (e.g., ICU preemption or enhanced monitoring) would prevent up to 42 unnecessary interventions per 100 patients compared to alternative strategies. The benefit plateaued beyond the 60% threshold, aligning with established clinical intervention criteria for high-risk immunocompromised populations. Calibration curve analysis (**Figure 6D**) confirmed that AutoML significantly outperformed other models in calibration, achieving the lowest Brier score (0.130) on the test set. For detailed metrics, see **Table 5** and **Figure 6**.

**Table 5:** Test set predictive performance metrics.

Models	PRE	SEN	SPE	ACC	F1	ROC-AUC	PR-AUC
LR	0.7241 (0.602-0.846)	0.8936 (0.795-0.992)	0.4667 (0.307-0.626)	0.7273 (0.620-0.835)	0.8000 (0.710-0.890)	0.7645 (0.651-0.878)	0.8041 (0.701-0.907)
SVM	0.6104 (0.486-0.735)	1.0000 (1.000-1.000)	0.0000 (0.000-0.000)	0.6104 (0.486-0.735)	0.7581 (0.664-0.852)	0.7312 (0.613-0.849)	0.7761 (0.668-0.884)
Adaboost	0.7167 (0.593-0.840)	0.9149 (0.825-1.000)	0.4333 (0.277-0.590)	0.7273 (0.620-0.835)	0.8037 (0.714-0.893)	0.7908 (0.683-0.899)	0.8377 (0.742-0.933)
XGBoost	0.7344 (0.615-0.854)	1.0000 (1.000-1.000)	0.4333 (0.277-0.590)	0.7792 (0.678-0.880)	0.8468 (0.770-0.924)	0.8369 (0.739-0.935)	0.8487 (0.755-0.942)
LightGBM	0.7708 (0.656-0.886)	0.7872 (0.657-0.917)	0.6333 (0.474-0.793)	0.7273 (0.620-0.835)	0.7789 (0.685-0.873)	0.8369 (0.739-0.935)	0.8847 (0.805-0.964)
AutoML	0.7925 (0.683-0.902)	0.8936 (0.795-0.992)	0.6333 (0.474-0.793)	0.7922 (0.694-0.890)	0.8400 (0.756-0.924)	0.9206 (0.854-0.987)	0.9273 (0.867-0.988)

To assess the robustness of the constructed AutoML prediction model across different patient subgroups, we conducted subgroup analyses stratified by transplant organ type. In the liver transplant subgroup (n=262), the AutoML model achieved an ROC-AUC of 0.915 (95% CI: 0.838-0.992) and a PR-AUC of 0.922 (95% CI: 0.852-0.992) on the independent test set (n=51). For the renal transplant subgroup (n=66), the model attained an ROC-AUC of 0.928 (95% CI: 0.803-1.000) and a PR-AUC of 0.938 (95% CI: 0.832-1.000) on its independent test set (n=15). Although the smaller sample size of the renal transplant subgroup resulted in wider confidence intervals, both subgroups maintained high model performance comparable to that in the overall population, indicating robust generalizability across transplant types.

### 3.6 Interpretability analysis

#### 3.6.1 LASSO regression analysis

**Figure 7:**
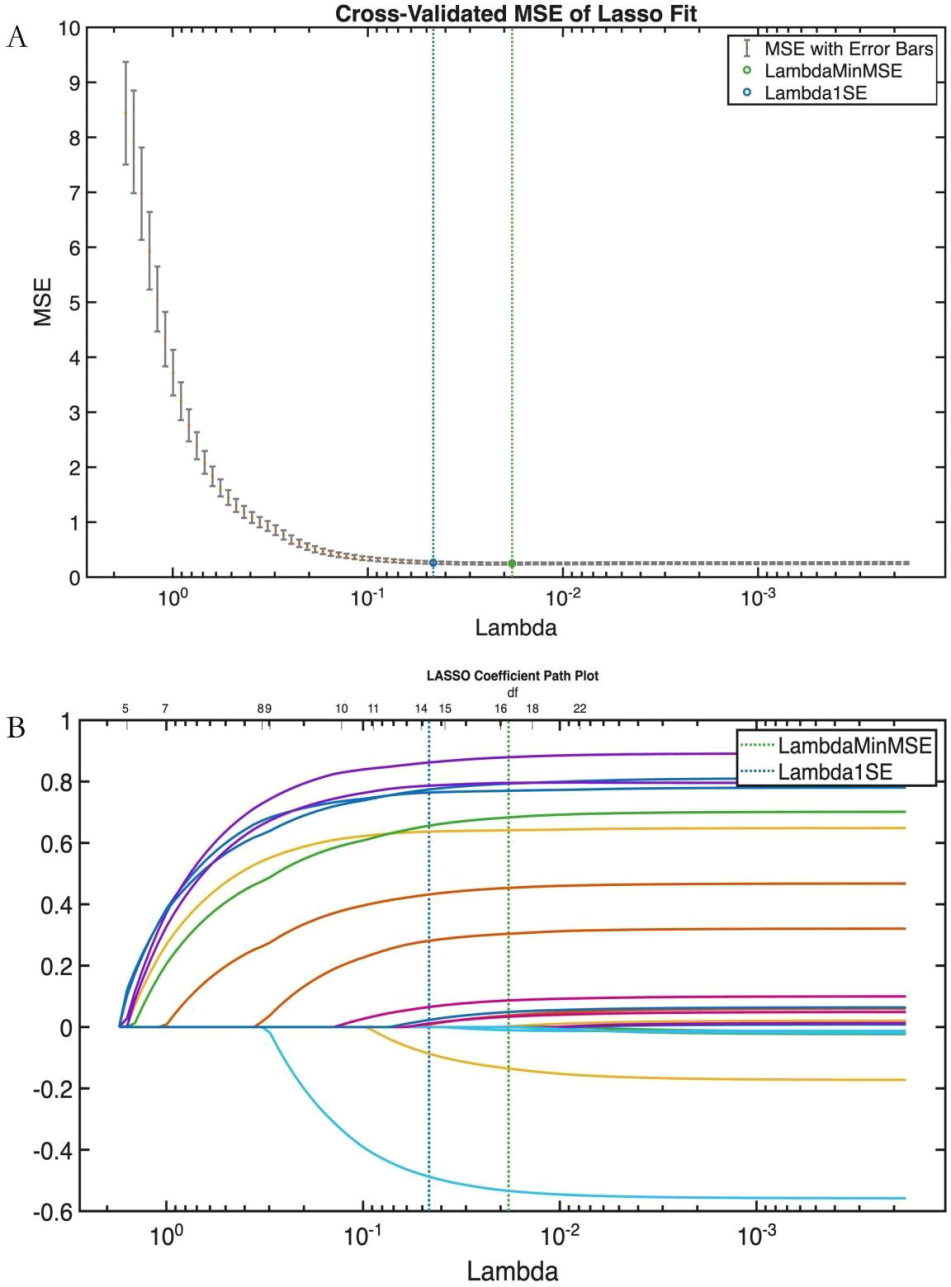
LASSO regression results. **(A)** LASSO trajectory plot; **(B)** LASSO cross-validation plot.

LASSO regression was applied to screen features in the training set (**Figure 7**), validating the effectiveness of AutoML feature selection. Using the Lambda1SE criterion (variables within one standard error of minimal MSE), LASSO identified 14 variables: mNGS bacterial detection, mNGS fungal detection, Procalcitonin (PCT), C-reactive protein (CRP), mNGS viral detection, white blood cell count, creatinine, post-transplantation time, neutrophil percentage, diabetes, age, total bilirubin, alanine aminotransferase (ALT), and lymphocyte percentage. The overlap rate with AutoML-selected features was 78.6% (11/14).

#### 3.6.2 SHAP analysis

**Figure 8:**
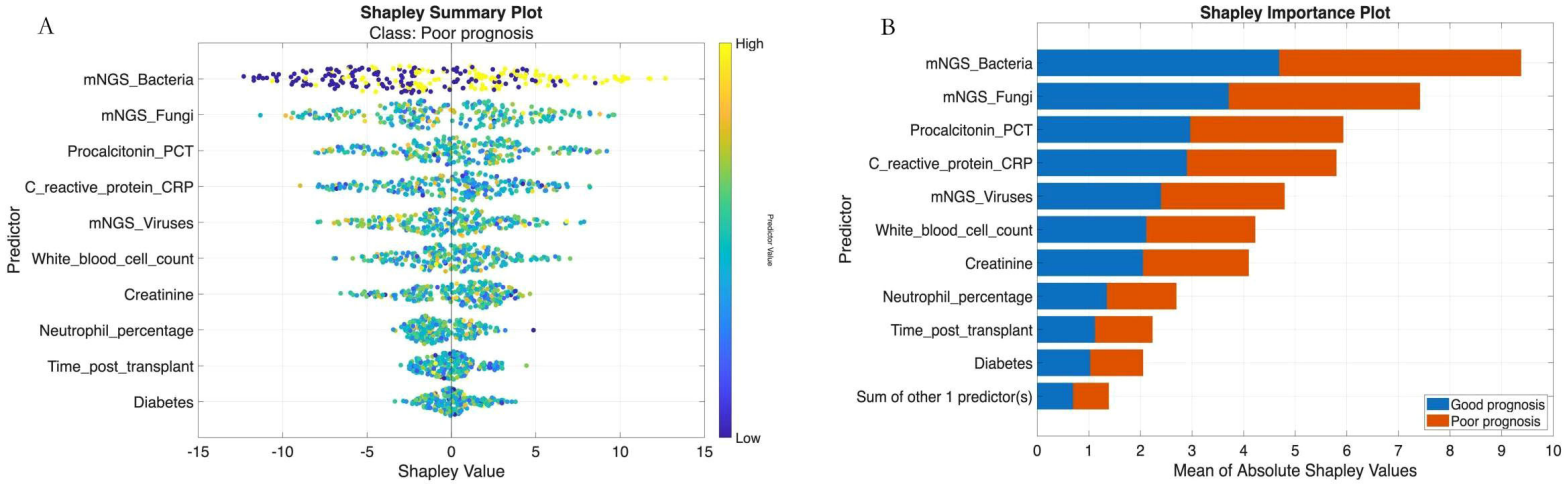
Global SHAP values of key predictors. **(A)** Shapley summary plot; **(B)** Shapley feature importance plot.

SHAP analysis ranked feature importance as follows: mNGS bacterial detection > mNGS fungal detection > PCT > CRP > mNGS viral detection > white blood cell count > creatinine > post-transplantation time > neutrophil percentage > diabetes > age (**Figure 8**).

**Figure 9:**
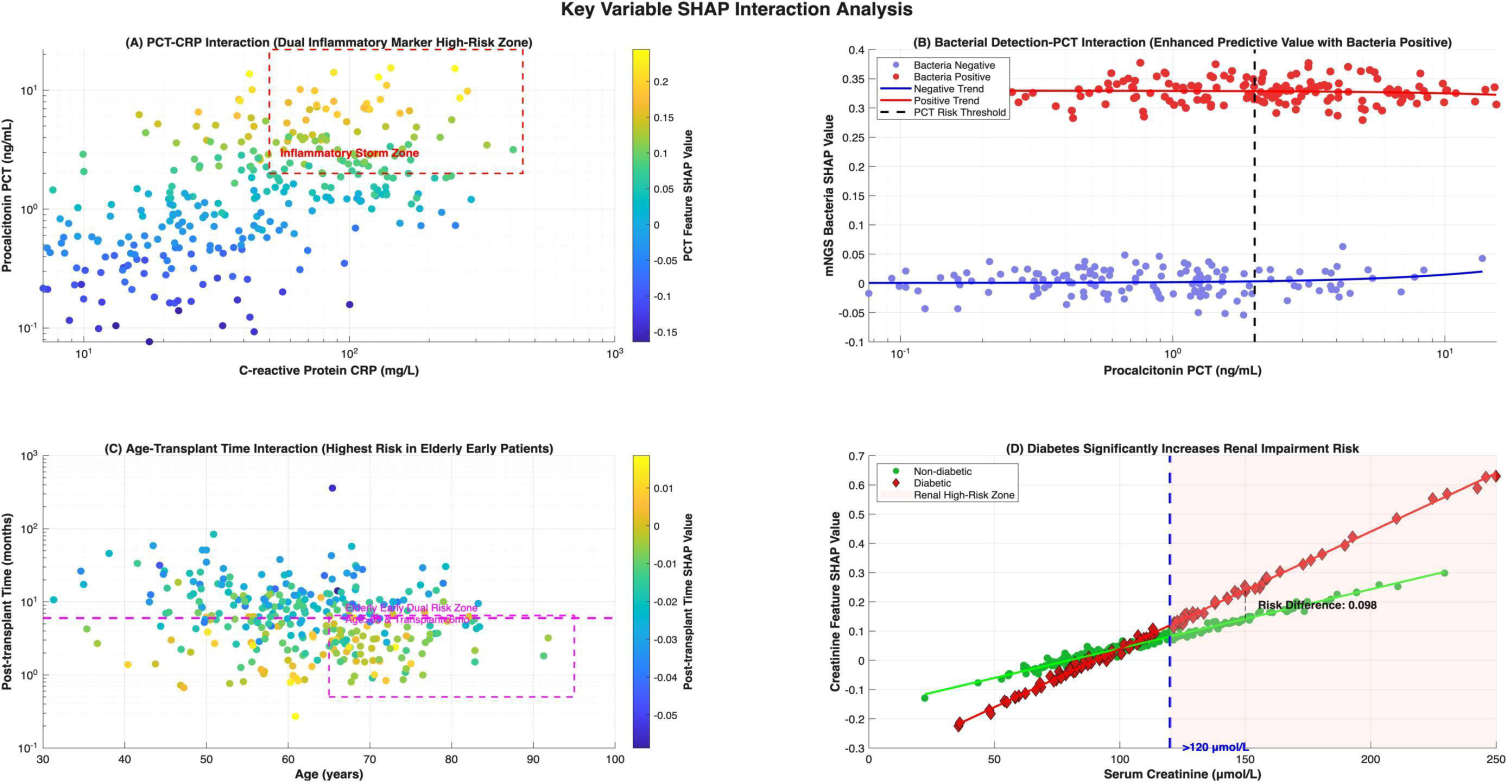
SHAP interaction analysis of key indicators.

Interaction analysis (**Figure 9**) revealed: (A) Inflammatory storm: CRP >50mg/L and PCT >2ng/mL concentrated in poor-prognosis subgroups. (B) PCT synergism: Bacterial positivity enhances PCT’s predictive value, doubling infection risk. (C) Dual high-risk: Age >65 years and transplantation time <6 months. (D) Diabetic nephropathy: Creatinine >120μmol/L in diabetic patients accelerates risk progression.

### 3.7 Visualization system demonstration

**Figure 10:**
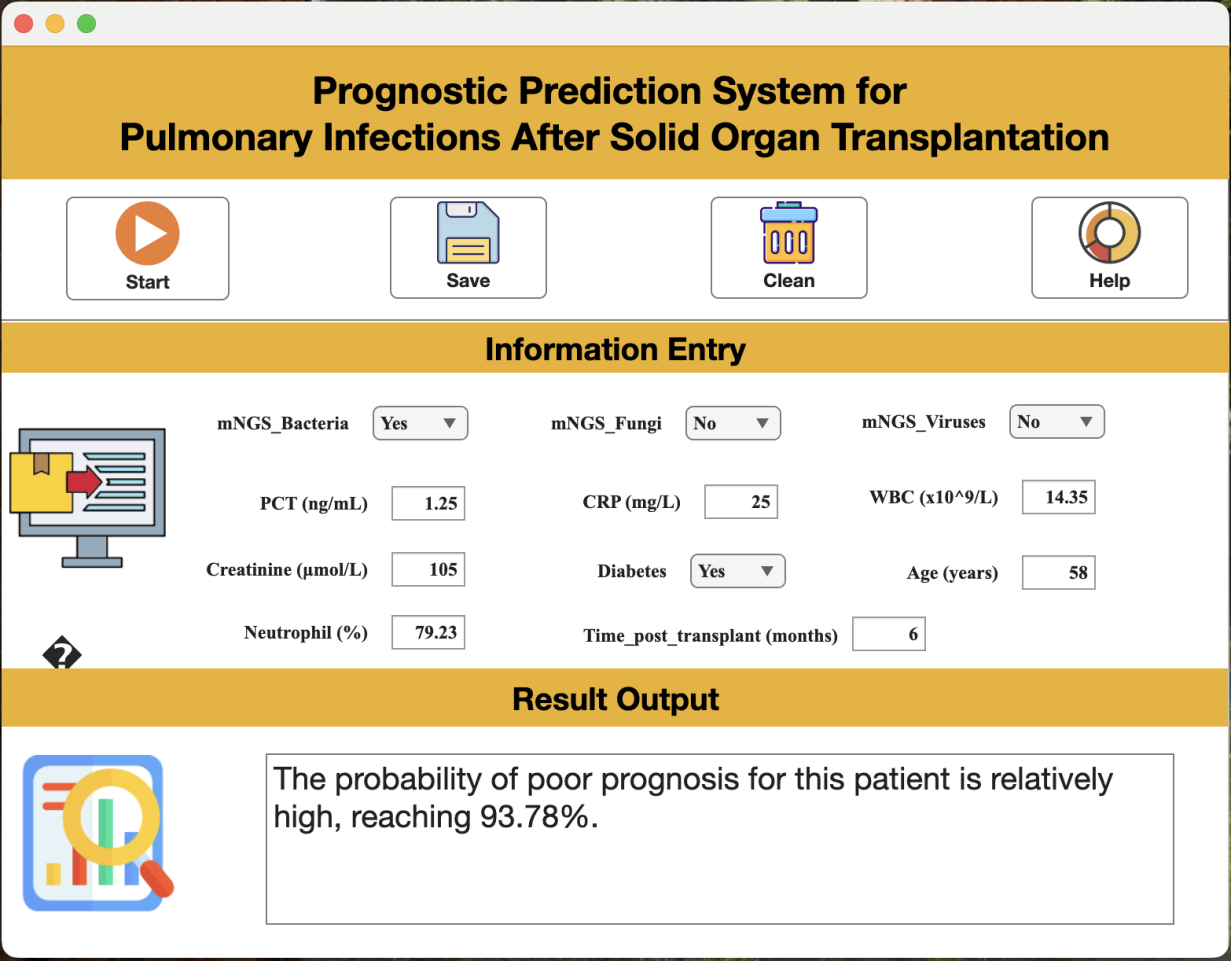
Software system operation demonstration.

The system features a visual interactive prediction interface. Clinicians input key indicators in the “Feature Input” panel, and the system calculates the probability of adverse outcomes (0-100%) in real-time using the trained AutoML model (**Figure 10**).

## 4 Discussion

Organ transplantation serves as an effective treatment for end-stage organ failure; however, postoperative pulmonary infections manifest as a major complication that significantly impacts patient and graft survival rates (11). Although routine clinical assessments (including clinical manifestations, laboratory tests, and imaging) can diagnose infections, they exhibit limitations in pathogen detection rates. This underscores the urgent need for rapid, accurate, and comprehensive diagnostic methods (12). As an emerging pathogen detection technology, metagenomic next-generation sequencing (mNGS) requires no predefined pathogen targets, remains unaffected by antibiotics, and directly performs nucleic acid extraction and sequencing on clinical samples. By identifying pathogenic microorganisms through bioinformatic alignment, mNGS demonstrates high sensitivity and short testing cycles in critical or pathogen-unidentified infections (13). Our results demonstrate that the overall positive detection rate of mNGS in identifying pathogens causing pulmonary infections post-solid organ transplantation significantly surpassed conventional methods, aligning with findings by Saadatzadeh T (14). This indicates substantially higher detection rates for viruses and bacteria in transplant recipients compared to traditional approaches, highlighting its broad clinical application prospects.

In this study, AutoML exhibited the strongest robustness in the independent test set, achieving ROC-AUC of 0.9206 and PR-AUC of 0.9273. Clinically, the model provides transformative incremental utility over conventional decision-making frameworks: (1) Versus physician judgment alone, the DCA confirmed benefit improvement in the 15%–60% risk range—a critical “clinical gray zone” where treatment decisions typically lack consensus. (2) Against existing prognostic scores, our model avoids their dependence on invasive/expensive parameters and leverages mNGS-derived microbial features for pathogen-specific risk stratification. However, operational barriers remain, including required informatics expertise for mNGS interpretation and cost constraints. Future development of tiered testing protocols and multicenter validation are prioritized to enhance real-world applicability. Calibration curve analysis confirmed that AutoML’s predictive calibration performance significantly outperformed other models. Its advantages stem from two key factors: AutoML automates feature selection, transformation, and generation, reducing overfitting by identifying core feature subsets, enhancing feature expression through standardization/normalization, and enriching the feature space through interaction terms to capture intrinsic data patterns. AutoML employs intelligent algorithms to automatically explore model spaces, utilizes meta-learning to identify optimal model architectures, and adopts strategies such as Bayesian optimization for hyperparameter tuning. This efficiently explores combinatorial possibilities to maximize performance, ensuring cross-dataset stability and accuracy (15, 16). While the AutoML model demonstrates potential for real-time risk stratification upon diagnostic data input, its clinical translation into interventional guidance requires prospective validation across diverse care settings. This qualification aligns explicitly with the limitations noted in our study—namely, single-center data constraints and absence of external validation cohorts.

Addressing limitations of traditional random forest algorithms in processing high-dimensional mNGS data for post-transplant pulmonary infection prediction, we propose an AutoML algorithm. This approach employs Tent chaotic mapping to enhance coverage of pathogenic feature parameter spaces, effectively capturing low-abundance pathogen signals, and utilizes dynamic Gaussian mutation mechanisms to significantly accelerate model convergence. Innovatively, our study integrates mNGS pathogen subtyping data (including bacterial/fungal/viral species and resistance gene profiles) with patient clinical characteristics (e.g., transplant organ type, postoperative time). This integration reveals non-linear associations between 11 core features—such as mNGS-detected bacteria/fungi, PCT, and CRP—and prognosis (17). SHAP analysis further confirmed the dominant role of pathogen load in risk prediction, particularly highlighting significantly elevated prognosis risks in patients with high pathogen loads combined with multidrug-resistant genes. This discovery provides a critical theoretical foundation for formulating individualized anti-infection regimens (18). While mNGS technology effectively overcomes limitations of conventional pathogen detection and enables rapid comprehensive pathogen profiling even in antibiotic-treated specimens, its turnaround time remains constrained by sequencing infrastructure requirements. Clinical implementation typically achieves results within 24–48 hours for critical cases, representing a reduction of 1–3 days compared to serial conventional testing in complex infections. However, the current feasibility threshold for routine first-line deployment is contingent upon specialized bioinformatics resources. In light of these operational considerations, we advocate a cascaded diagnostic approach: Routine screening should prioritize conventional methods (cultures/PCR) for cost efficiency; mNGS deployment should be strategically reserved for: Culture-negative cases, suspected rare/fastidious pathogens, immunocompromised patients with deteriorating conditions. Notably, our AutoML framework circumvents time-delay concerns during clinical deployment by leveraging pre-trained parameters for instant risk calculation upon data input (including mNGS results), generating real-time prognostic probabilities within the decision support system. This balances diagnostic precision with clinical pragmatism.

Our study, employing dual-dimensional validation through LASSO regression and SHAP interpretability analysis, identified 14 variables that demonstrate significant associations with prognosis in related pathophysiological mechanisms. Analysis indicates the underlying reasons as follows: Pathogen-Specific Factors, mNGS-detected pathogens (bacteria/fungi/virus) directly identify pathogen types causing infection. Detection of highly pathogenic or multidrug-resistant pathogens substantially increases the difficulty of anti-infection therapy, directly contributing to worsened prognosis. Conversely, precise pathogen identification guides targeted treatment, thereby improving prognosis (19). Inflammatory Response Markers, PCT and CRP, as classic inflammatory markers, directly correspond to infection severity. Persistently high levels indicate uncontrolled inflammation, potentially triggering complications like sepsis and reducing treatment efficacy (20). Immune System Indicators, Elevated white blood cell count and neutrophil percentage reflect the body’s stress response against infections, with both abnormally high or low values indicating immune imbalance. Decreased lymphocyte percentage suggests excessive post-transplant immunosuppression, impairing pathogen clearance and directly affecting prognosis (21). Organ Function Parameters, Creatinine, total bilirubin, and ALT reflect renal and hepatic function. Post-transplant patients inherently face organ functional burdens, while infections further aggravate liver/kidney injuries. Organ dysfunction concurrently restricts anti-infective drug selection and dosage, creating a vicious cycle that worsens prognosis (22). Baseline Health Conditions, Age and diabetes represent essential health determinants. Elderly patients experience natural immune decline, while diabetics exhibit vascular complications and immune dysregulation—both conditions reduce infection resistance, increasing susceptibility to severe complications and poorer prognosis (23). Peri-Transplant Vulnerability, Early post-transplant patients receive higher immunosuppressant doses with the weakest immunity. Pulmonary infections during this phase facilitate pathogen dissemination, leading to significantly higher prognostic risks than later stages (24). Key Interactive Findings on Prognosis Interpretation: mNGS Pathogens & Inflammatory Markers (SHAP Interaction), Bacterial detection + PCT synergy: When bacteria are confirmed by mNGS, PCT’s SHAP value increases significantly—a finding clinically consistent with pathophysiology: bacterial components (e.g., lipopolysaccharides) activate inflammatory pathways, amplifying PCT synthesis. This “pathogen-inflammation amplification” synergy promotes organ damage. Reduced impact: Absent bacterial detection by mNGS, mild PCT elevation minimally affects prognosis (near-zero SHAP interaction value). Age & Neutrophil Interaction for Risk Stratification, Patients >60 years with neutrophil % >80% show markedly higher SHAP values than younger counterparts—elderly patients exhibit impaired pathogen clearance, rendering high neutrophils an indicator of “immune exhaustion” that compounds risk. Younger patients: Neutrophil elevation typically reflects active infections and SHAP values decrease rapidly post-treatment, implying reversible prognostic impact.

### 4.1 Limitations and future perspectives

Limitations: Single-center bias: Models primarily rely on single-center data lacking multicenter/external validation by large samples, impeding generalization across clinical settings (center-specific protocols/care quality may degrade performance). The single-center retrospective design introduces inherent biases requiring explicit acknowledgment and contextualization. Selection bias arises from exclusively including post-transplant pulmonary infection patients who underwent mNGS testing. While clinically indicated, this criterion may exclude immunocompromised hosts with atypical presentations or resource-constrained settings lacking mNGS access, potentially limiting generalizability to broader transplant populations. Information bias stems from retrospective data acquisition, where incomplete documentation (e.g., unrecorded immunosuppressant levels or nuanced clinical assessments) could affect variable completeness. We mitigated this via rigorous multi-source validation (laboratory systems, nursing records, imaging databases) and clinical logic-based imputation. Unmeasured confounders (e.g., socioeconomic status, granular environmental exposures, or undocumented outpatient interventions) may influence both pathogen profiles and prognostic outcomes. Our model’s development on available multidimensional data partially offsets this, yet residual confounding remains possible. Future multicenter collaborations leveraging standardized protocols will be essential to address these limitations. mNGS interpretation complexity: Vast data volume demands bioinformatic/statistical expertise, challenging non-specialist interpretation while data decoding accuracy remains suboptimal. Cost constraints: Relatively high mNGS costs limit widespread adoption, especially for economically disadvantaged patients and resource-limited institutions. Unstandardized protocols: Variable analytic workflows/lack of harmonized standards cause inter-laboratory result variability, undermining reliability and reproducibility. Model generalizability: Predictive accuracy may suffer from multiple confounders (sample size, patient heterogeneity, data completeness), hindering patient-specific prognostic applications. While the test set performance demonstrates promising discriminative ability, we acknowledge two critical constraints: The moderate sample size (n=328) relative to predictor variables limits model complexity tolerance. Although our calculation confirmed an EPV of ∼6.5 for the training set (72 events/11 variables)—exceeding the minimal EPV≥5 threshold but falling short of the ideal EPV≥10—this may affect stability in broader populations. External validation is currently lacking, meaning the model’s generalizability to heterogeneous cohorts (e.g., differing geographic regions, transplant protocols, or pathogen profiles) remains untested. These factors warrant caution against immediate clinical deployment without further multicenter verification.

Future Directions: Establish multicenter platforms: Collaborate with geographically/institutionally diverse centers (e.g., transplant hospitals, multispecialty centers) to unify inclusion/data collection standards and curate large-scale external validation datasets to mitigate center-specific biases. Collaborations with geographically diverse transplant centers will standardize data collection (e.g., immunosuppression regimens, mNGS workflows) and curate large-scale cohorts. This framework will robustly assess model transportability across institutions with varying resource availability and patient heterogeneity. Dynamic pathogen database: Integrate genomic features of common post-transplant pathogens (CMV, Pneumocystis, resistant bacteria) with clinical diagnostics to optimize annotation algorithms and enhance interpretation accuracy. Tiered testing strategy: Prioritize conventional pathogen detection (sputum culture, PCR); reserve mNGS for negative cases or suspected rare infections to reduce costs and improve accessibility in resource-limited settings. Inter-laboratory quality control: Implement standardized EQA schemes with reference samples to align cross-institutional results and boost reproducibility. Dynamic risk prediction: Leverage serial postoperative monitoring data (e.g., weekly mNGS/monthly immune function) to update model parameters in real-time and generate personalized risk curves for early detection of worsening infections/poor prognosis.

## 5 Conclusion

The prognostic prediction model developed in this study integrates clinical data, machine learning, and decision support. Its core value extends beyond surpassing traditional models’ predictive power—it pioneers an interpretable, user-friendly, and high-efficacy risk assessment paradigm, opening novel pathways for improving pulmonary infection outcomes after solid organ transplantation.

## Data Availability

The raw data supporting the conclusions of this article will be made available by the authors, without undue reservation.
